# Structural insights into a broadly reactive nanobody that binds pathogenic and non-pathogenic lagoviruses

**DOI:** 10.1128/jvi.01990-25

**Published:** 2026-01-08

**Authors:** Derin Kara, Marie Pancera, Gholamreza Hassanzadeh-Ghassabeh, Steve Schoonooghe, Veronika Masic, Lauren Hartley-Tassell, Biswa Prasanna Mishra, Thomas Ve, Thomas Haselhorst, Mark von Itzstein, Grant S. Hansman

**Affiliations:** 1 Vaccine and Infectious Disease Division, Fred Hutchinson Cancer Center 7286, Seattle, Washington, USA; 2 VIB Nanobody VHH Core, Vrije Universiteit Brussel 70493, Brussels, Belgium; 3 Institute for Biomedicine and Glycomics, Griffith University, Gold Coast Campus, Gold Coast, Queensland, Australia; St Jude Children's Research Hospital, Memphis, Tennessee, USA

**Keywords:** nanobody, X-ray crystallography, calicivirus

## LETTER

Pathogenic genogroup I rabbit hemorrhagic disease virus (RHDV) is genetically related to non-pathogenic GII hare calicivirus (HaCV) in the *Lagovirus* genus (1). Lagovirus capsid protruding (P) domains, analogous to human noroviruses, engage histo-blood group antigens (HBGAs) as co-factors (2–10). In this study, we generated nanobody libraries against HaCV and RHDV-K5 (a biocontrol agent released in Australia in 2017) (11, 12) virus-like particles (VLPs) to identify nanobodies with broad reactivity.

**Fig 1:**
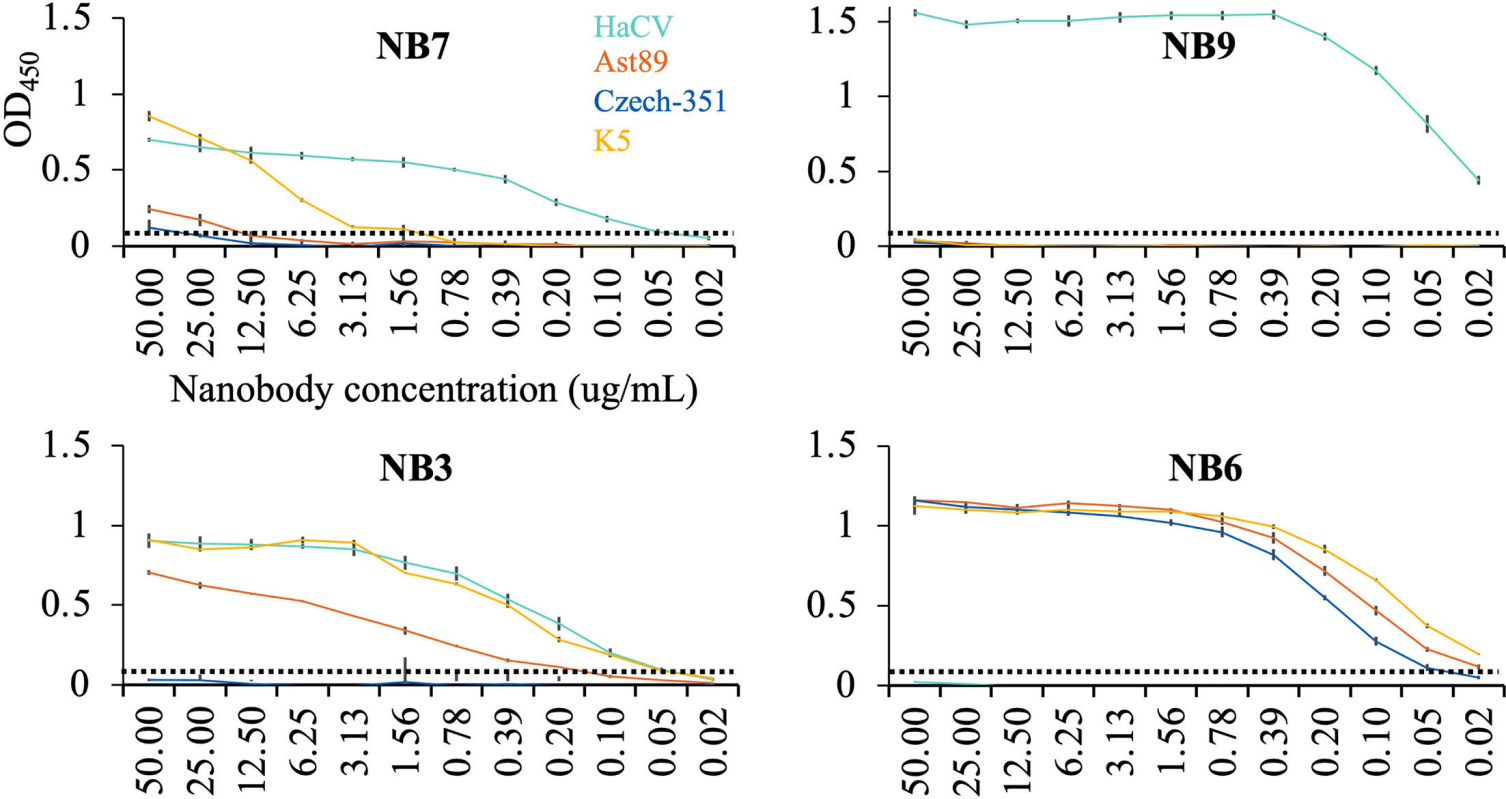
Development of lagovirus nanobodies. Cross-reactivity was assessed by direct ELISA (13) using VLPs (0.02 µg/mL) of HaCV (GenBank ID MK138384.1), Ast89 (Z49271), Czech-351 (KF594473.1), and K5 (MF598301.1). Nanobodies were serially diluted in PBS and detected with an anti-His HRP-conjugated monoclonal antibody. Error bars indicate triplicate wells, and the dashed line denotes the binding cutoff at an optical density (OD_450_) of 0.05 with an error bar shown for three replicates (14–16). NB7 and NB9, raised against HaCV, reacted to HaCV, with NB7 also cross-reacting with K5 and weakly cross-reacting to Czech-351 and Ast89. NB3 and NB6, raised against K5, bound K5, while NB3 additionally recognized HaCV and Ast89, whereas NB6 cross-reacted with Ast89 and Czech-351.

Two HaCV nanobodies (NB7 and NB9) and two K5 nanobodies (NB3 and NB6) were expressed and evaluated for cross-reactivity with HaCV, K5, RHDV-Ast89, and RHDV-Czech-351 (a biocontrol agent released in Australia in 1996) (11, 12) VLPs using a direct ELISA (Fig. 1) (13). NB7 bound HaCV (0.05 µg/mL), cross-reacted with K5 (3.13 µg/mL), and showed weak cross-reactivity to both RHDV Ast89 and Czech-351 (25–50 µg/mL). NB9 bound HaCV (<0.02 µg/mL) and showed no cross-reactivity to the three RHDVs. NB3 bound K5 (0.05 µg/mL), cross-reacted with both HaCV (0.05 µg/mL) and Ast89 (0.2 µg/mL), but not Czech-351. NB6 bound K5 (<0.02 µg/mL), cross-reacted with both Ast89 and Czech-351 (<0.02 µg/mL), and had no cross-reactivity to HaCV.

**Fig 2:**
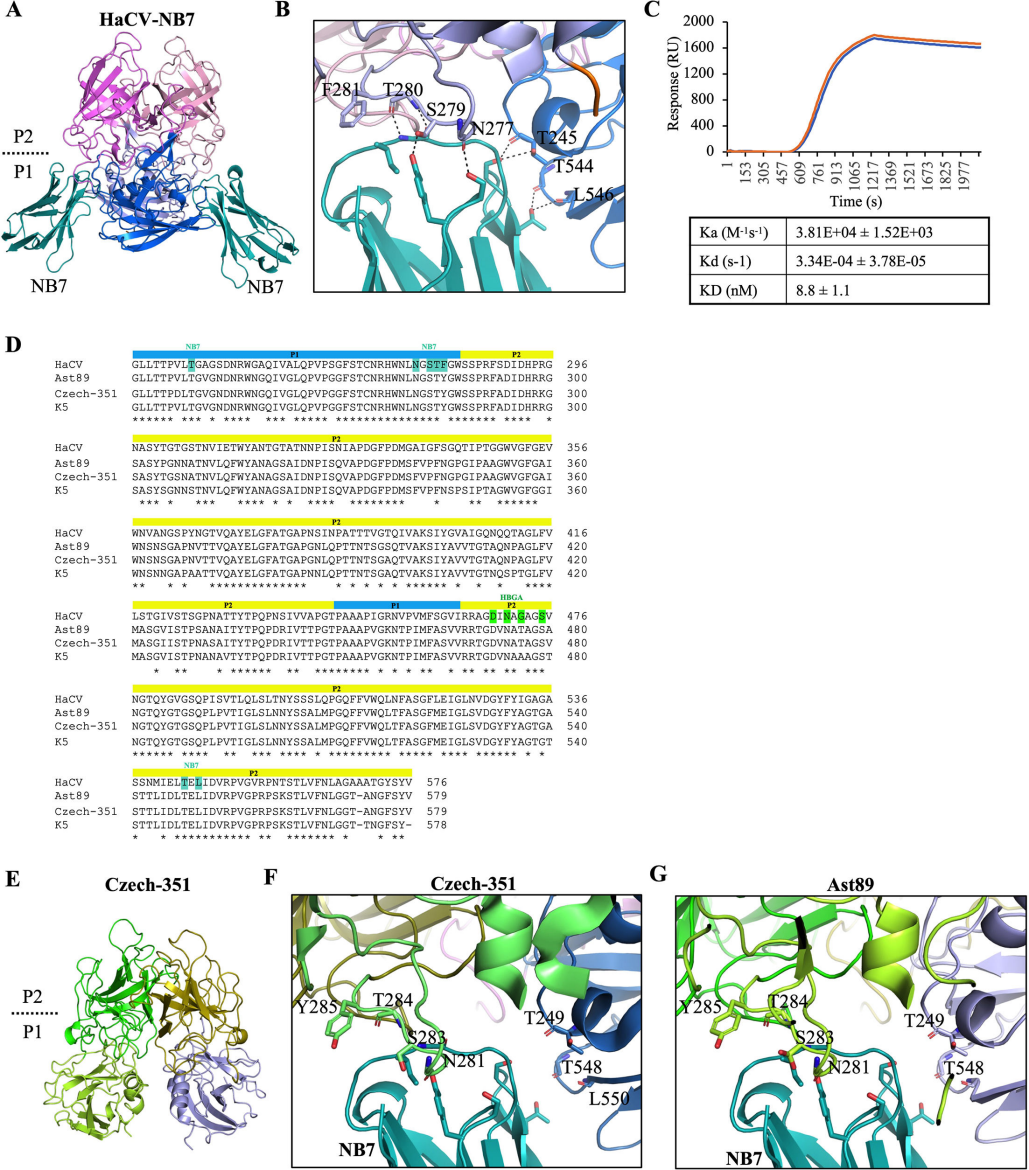
HaCV P domain and NB7 binding interactions. (**A**) The X-ray crystal structure of the HaCV-NB7 complex was processed using XDS (21) at 2.09 Å resolution. The complex was solved by molecular replacement in PHASER (22) using Nano-85 (PDB ID 4X7E) and HaCV-apo (PDB ID 9DR0) as search models and refined in multiple rounds of manual model building in COOT (23) and PHENIX (24). The HaCV P domain is colored by structural region: shell (residues 233–234: orange^chain A/B^), P1 subdomain (residues 235–283, 446–463, and 480–576: light blue^chain A^ and marine^chain B^), P2 subdomain (residues 284–445 and 464–479: light pink^chain A^ and violet^chain B^), and NB7 (teal). Two NB7 molecules bound to the HaCV P domain dimer (gray), subdivided into P1 and P2 subdomains. (**B**) Binding interfaces and interactions were analyzed using PyMOL (version 2.1) with hydrogen bonds and water-mediated interaction distances less than ~3.2 and ~3.5 Å, respectively. The close-up of one NB7 molecule binding to the HaCV P domain, where both side chains (S279^chain A^ and T245^chain B^) and main chain (N277^chain A^, T280^chain A^, T245^chain B^, T544^chain B^, and L546^chain B^) residues contributed to NB7 binding. A hydrophobic interaction was provided by the side chain F281, and numerous water-mediated interactions were observed (not shown). A similar interaction was observed with the other NB7 molecule. (**C**) Binding kinetics was determined using a Sartorius SF3 SPR instrument and a His-cap sensor, where His-tagged NB7 was captured onto the surface of the sensor. The sensor was prepared by a 60 s injection at 10 µL/min of 0.5 mM NiSO_4_. NB7 was diluted in PBS and flowed over the activated surface for 180 s at a flow rate of 5 µL/min. The HaCV P domain was diluted to 1 µM and was injected across the surface using the OneStep injection method with 100% of loop volume at a flow rate of 50 µL/min with a 280 s dissociation step. The surface was regenerated by an injection of 350 µM EDTA for 60 s at 10 µL/min and 100 mM imidazole for 60 s at 40 µL/min. Buffer-only controls were also run every second cycle to enable double reference subtraction of the data. The data generated were from four replicates, and duplicates (blue and orange) are shown on the graph. (**D**) Amino acid alignment of HaCV, Ast89, Czech-351, and K5 partial capsid sequences, where the blue and yellow bars represent the P1 and P2 subdomains, respectively. The teal and green boxes represent HaCV P domain residues that bind NB7 and HBGA (A-trisaccharide), respectively (20). (**E**) X-ray crystal structure of Czech-351-apo was determined at 1.37 Å resolution. The Czech-351 P domain is colored by subdomain: P1 subdomain (residues 231–286, 450–466, and 484–579: limon^chain A^ and light blue^chain B^) and P2 subdomain (residues 287–449 and 467–483: green^chain A^ and olive^chain B^). HaCV-NB7 (showing only NB7) superimposed on (**F**) Czech-351-apo (colored as in panel **E**) and (**G**) Ast89-apo is colored by subdomain: P1 subdomain (residues 231–286, 450–466, and 484–579: lime^chain A^ and sky blue^chain B^) and P2 subdomain (residues 284–445 and 464–479: deep olive^chain A^ and violet^chain B^) show NB7 equivalent residues with a root mean square deviation = 0.578 Å (590 Cα atoms) and 0.623 Å (600 Cα atoms), respectively.

**Fig 3:**
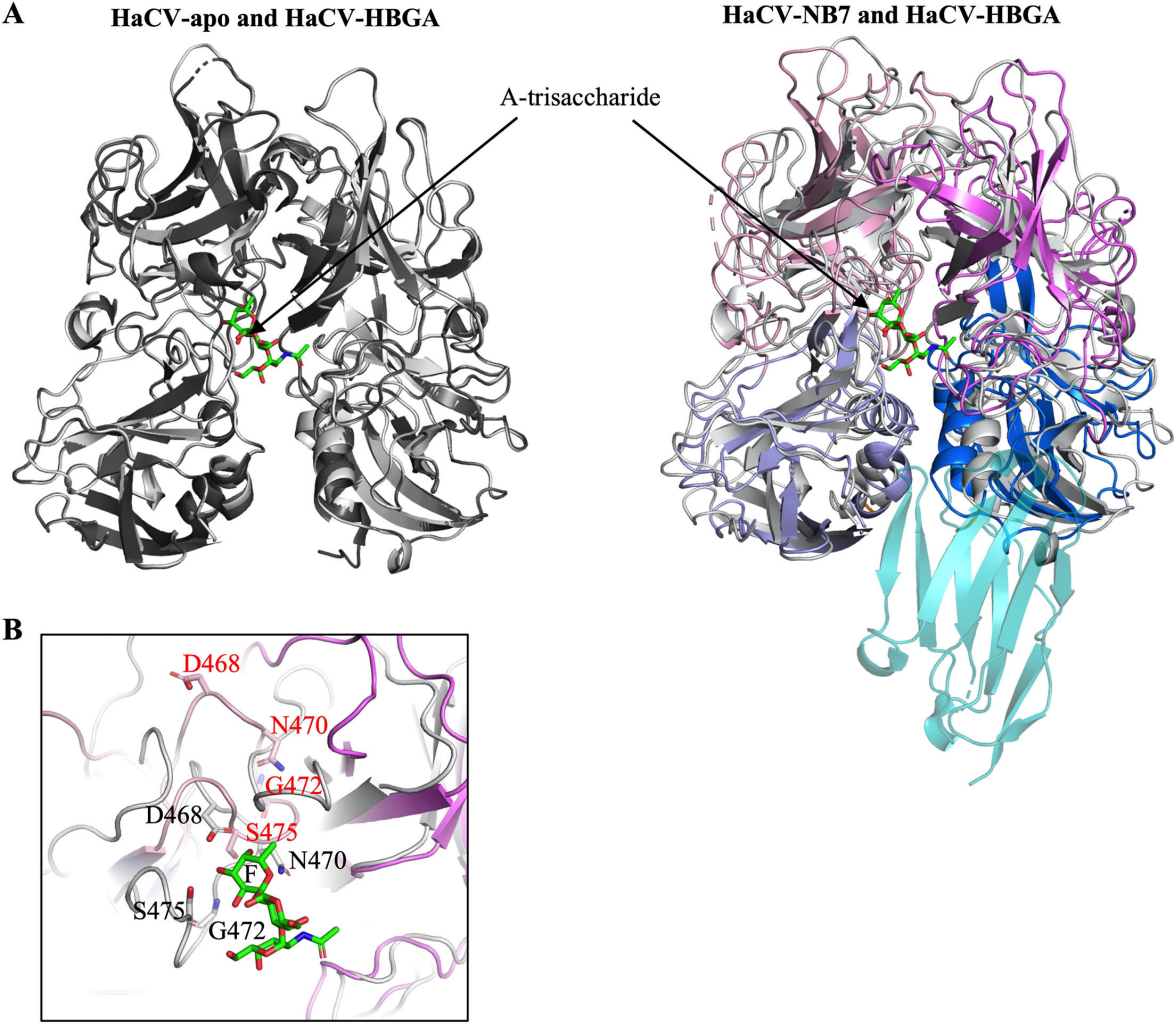
NB7 modulates distal loops to reshape the P domain. (**A**) Superposition of HaCV-apo (black) and the HaCV-HBGA A-trisaccharide complex (gray and green sticks^A-trisaccharide^) (PDB ID 9DQC) P domain dimers (chains A and B) show they are highly very similar and well aligned with a root mean square deviation (RMSD) = 0.095 Å (587 Cα atoms, aligned/calculated in PyMOL), whereas superposition of HaCV-HBGA and HaCV-NB7 P domain dimers show numerous P domain loop movement and RMSD = 2.805 Å (580 Cα atoms). (**B**) Close-up view of the HBGA binding pocket with a superposition of the HaCV HBGA A-trisaccharide complex (gray) and the HaCV-NB7 complex (colored as in Fig. 2A). Residues involved in A-trisaccharide binding are labeled in black, while the corresponding residues in the HaCV-NB7 complex are labeled in red, highlighting the extent of loop movements within the HBGA pocket. The fucose moiety of the A-trisaccharide is labeled “F.”

Crystallization trials with the corresponding HaCV, Ast89, K5, and Czech-351 P domains yielded crystals only for the HaCV P domain and the NB7 complex (HaCV-NB7) (Table 1). Structural analysis showed that NB7 recognized a conformational epitope at the bottom of the HaCV P domain, where it interacted with residues from both monomers at the dimeric interface (Fig. 2A and B). Interestingly, the overall NB7 binding site on the HaCV P domain was similar to several broadly reactive diagnostic and therapeutic norovirus nanobodies (14–19). Affinity measurements found that NB7 bound strongly to the HaCV P domain (KD = 8.8 nM) (Fig. 2C). An amino acid sequence alignment revealed that the residues involved in NB7 binding are highly conserved among RHDVs (Fig. 2D). To obtain further structural insight into the NB7 conformational binding epitope, we determined the Czech-351-apo structure (Fig. 2E and Table 1) and, together with the Ast89-apo structure (PDB ID: 8CYL), performed superimpositions with the HaCV-NB7 and HaCV-apo structures. Most of the corresponding Czech-351 (Fig. 2F) and Ast89 (Fig. 2G) P domain loops and residues predicted to interact with NB7 aligned closely with the HaCV-apo structure, indicating the conformational NB7 epitope was preserved. However, structural comparison of HaCV-apo, HaCV-HBGA, and HaCV-NB7 revealed that NB7 binding induced extensive P domain loop rearrangements (Fig. 3A), including residues that bound NB7 and residues that engaged the HBGA (Fig. 3B) (20). Although certain P domain loop rearrangements in the HaCV-NB7 complex may be influenced by crystal lattice contacts, our extensive structural studies with norovirus P domain nanobody complexes show that nanobody binding did not result in substantial conformational changes in the P domain loops (14–19).

**TABLE 1:** Data collection and refinement statistics of P domain X-ray crystal structures

	HaCV-NB7	Czech
Data collection		
Space group	C 1 2 1	P 1 21 1
Cell dimensions		
*a*, *b*, *c* (Å)	214.25, 73.51, 59.25	62.968, 51.261, 94.27
*α*, *β*, *γ* (°)	90.0, 94.11, 90.0	90.0, 101.051, 90.0
Resolution range (Å)	29.66–2.09 (2.14–2.09)	47.37–1.37 (1.39–1.37)
No. of unique reflections	53,856 (3903)	124,329 (5870)
*R*_merge_*^a^*	0.103 (0.754)	0.066 (0.900)
*R*_meas_^*b*^	0.115 (0.843)	0.072 (0.991)
*R*_pim_^*c*^	0.050 (0.371)	0.030 (0.408)
<*I*/*σ*(*I*)>	10.7 (1.9)	11.3 (1.3)
CC_1/2_	0.998 (0.667)	0.999 (0.666)
Completeness	98.8% (96.9)	99.6% (95.8)
Multiplicity	5.0 (5.0)	5.7 (5.6)
Refinement		
Resolution range (Å)	29.66–2.09 (2.17–2.09)	47.37–1.37 (1.42–1.37)
*R*_work_/*R*_free_^*d*^	18.16/22.27 (24.81/30.22)	14.99/17.12 (23.62/25.56)
No. of atoms	6,812	5,808
Protein	6,447	4,929
Water	365	807
Ligand	0	72
B-factors (Å^2^)	40.00	17.39
Protein	40.25	15.51
Water	35.73	27.67
Ligand	N/A	30.37
RMS bond length (Å)	0.008	0.012
RMS bond angle (°)	0.89	1.23
Ramachandran plot statistics^*e*^		
Residues	871	669
Most favored region	96.50	97.14
Allowed region	3.27	2.86
Disallowed region	0.23	0.00
Clashscore	4.19	2.53
PDB ID	9YP2	9YP1
Mother solution	0.2 M sodium fluoride and 20% PEG3350	0.1 M CHES (pH 9.0) and 20% PEG8000

^*a*^ *R*_merge _= Σ_h _Σ_*i*_|*I*_(*h*)*i*_– *I*_*h*_| / Σ_*h*_Σ_*i*_*I*_(*h*)*i*_, where *I*_h _is the averaged intensity of all reflections *h*.

^*b*^ *R*_meas _= Σ_*h*_[*N */ (*N *– 1)]^1/2 ^Σ_*i*_|*I*_(ih) _– *I*_*h*_| / Σ_*h*_Σ_*i*_*I*_(ih)_.

^*c*^ *R*_pim _= Σ_*h*_[1 / (*N *– 1)]^1/2 ^Σ_*i*_|*I*_(ih) _– *I*_h_| / Σ_*h*_Σ_*i*_*I*_(ih)_.

^*d*^ *R*_work _and *R*_free _= ∑|*F*_obs _– *F*_calc_| / ∑|*F*_obs_| × 100 for 95% of recorded data (*R*_work_) or 5% data (*R*_free_).

^*e*^ Determined using MolProbity.

This new finding of NB7 binding highlights a novel structural plasticity of the calicivirus P domain and a possible allosteric interference at the HBGA pocket. Future work is expected to test the potential of NB7 to inhibit lagovirus binding to HBGAs and explore how these nanobodies perform in diagnostic assays.
